# Prion-like spreading of Alzheimer’s disease within the brain’s connectome

**DOI:** 10.1098/rsif.2019.0356

**Published:** 2019-10-16

**Authors:** Sveva Fornari, Amelie Schäfer, Mathias Jucker, Alain Goriely, Ellen Kuhl

**Affiliations:** 1 Living Matter Laboratory, Stanford University, Stanford, CA, USA; 2 Hertie-Institute for Clinical Brain Research/German Center for Neurodegenerative Diseases, Tübingen, Germany; 3 Mathematical Institute, University of Oxford, Oxford, UK

**Keywords:** neurodegeneration, Alzheimer’s disease, prion, network, connectome, graph Laplacian

## Abstract

The prion hypothesis states that misfolded proteins can act as infectious agents that template the misfolding and aggregation of healthy proteins to transmit a disease. Increasing evidence suggests that pathological proteins in neurodegenerative diseases adopt prion-like mechanisms and spread across the brain along anatomically connected networks. Local kinetic models of protein misfolding and global network models of protein spreading provide valuable insight into several aspects of prion-like diseases. Yet, to date, these models have not been combined to simulate how pathological proteins multiply and spread across the human brain. Here, we create an efficient and robust tool to simulate the spreading of misfolded protein using three classes of kinetic models, the Fisher–Kolmogorov model, the Heterodimer model and the Smoluchowski model. We discretize their governing equations using a human brain network model, which we represent as a weighted Laplacian graph generated from 418 brains from the Human Connectome Project. Its nodes represent the anatomic regions of interest and its edges are weighted by the mean fibre number divided by the mean fibre length between any two regions. We demonstrate that our brain network model can predict the histopathological patterns of Alzheimer’s disease and capture the key characteristic features of finite-element brain models at a fraction of their computational cost: simulating the spatio-temporal evolution of aggregate size distributions across the human brain throughout a period of 40 years takes less than 7 s on a standard laptop computer. Our model has the potential to predict biomarker curves, aggregate size distributions, infection times, and the effects of therapeutic strategies including reduced production and increased clearance of misfolded protein.

## 1. Motivation

A major advance in our understanding of the human brain has been the realization that the our brain is organized as a network, both at the physical and at the functional levels [1]. This quiet revolution has been made possible by the parallel development of network theory and medical imaging, in particular by the concept of small-world networks [2]. Methods originating from graph theory are now routinely used to study many aspects of brain function and the prevalent dogma is that the brain operates as an efficiently structured, modular, dynamic network with strongly connected hubs [3]. This network is optimized to rapidly transmit information, but, unfortunately, the concept of fast transport also applies to misfolded proteins that highjack the network to rapidly spread across the brain [4]. Previous work has shown that the eigenmodes of the brain network’s graph Laplacian are correlated to brain atrophy in Alzheimer’s disease [5], and probablistic epidemiological models have been proposed to study transference mechanisms within the network [6].

**Figure 1.**
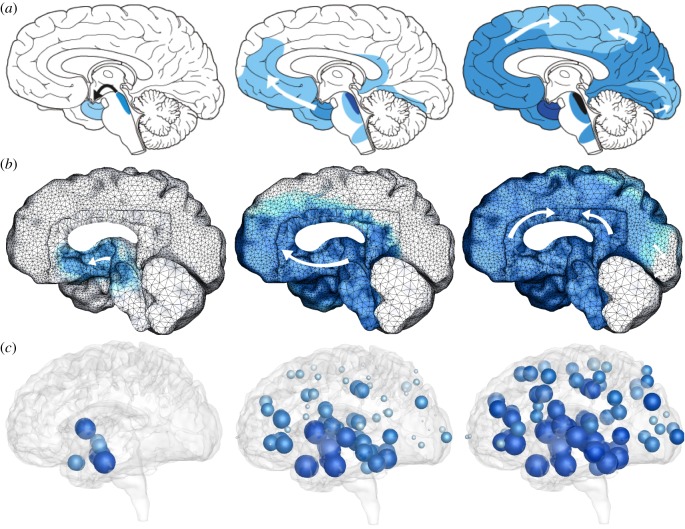
Typical pattern of tau protein misfolding in Alzheimer’s disease. (*a*) Clinical observation [15], (*b*) continuum model [16] and (*c*) network spreading model display characteristic pattern with misfolded tau occurring first in the locus coeruleus and transentorhinal layer from where they spread to the transentorhinal region and the proper entorhinal cortex and ultimately affect all interconnected neocortical brain regions. (Online version in colour.)

The current prevalent theory for neurodegenerative diseases is based on the prion-like paradigm [7] in which neurodegeneration is caused by the systematic invasion and conformational autocatalytic conversion of misfolded proteins [8]. In Alzheimer’s disease, Amyloid beta and tau proteins are believed to act in a prion-like manner and misfold [9]. This misfolded form of the protein acts as a template on which healthy proteins misfold and grow into increasingly larger aggregates [10]. Amyloid beta is an extracellular protein that mainly spreads across the extracellular matrix [11], whereas tau is an intracellular protein that primarily propagates within the network of axonal pathways [12]. Here, we focus on tau, which spreads across the brain in a highly predictable pattern [13]: misfolded tau proteins occur first in the locus coeruleus and the transentorhinal layer from where they spread to the transentorhinal region and the proper entorhinal cortex and ultimately affect all interconnected neocortical brain regions [14]. Figure 1*a* illustrates the typical spatio-temporal pattern of misfolded tau protein in Alzheimer’s disease inferred from histopathological observations of hundreds of human brains [15].

Understanding the progression of Alzheimer’s disease is a matter of understanding the physical processes of misfolding and transport. From a modelling perspective, three approaches have been proposed to simulate the physics of neurodegeneration: (i) kinetic growth and fragmentation models to study the local interaction of aggregates of different sizes using a set of ordinary differential equations [17]; (ii) network diffusion models to study the global prion-like spreading of misfolded proteins using graph theory [5]; and (iii) reaction–diffusion-based continuum models to study the spatio-temporal evolution of patheogenic proteins using partial differential equations [18].

Figure 1*b* shows that continuum models with nonlinear reaction and anisotropic diffusion can accurately predict the typical pattern of tau protein misfolding in Alzheimer’s disease [16]. This simulation used a Fisher–Kolmogorov model [19,20], discretized with 400 000 tetrahedral finite elements and 80 000 d.f. The continuum model displays an excellent agreement with clinical observations. However, it is computationally expensive and impractical to systematically explore a wide variety of disease and treatment scenarios. In addition, there is currently no technology to validate its predicted spreading patterns at a high enough resolution that would truly warrant a finite-element simulation with thousands of degrees of freedom. The objective of this study is therefore to create an efficient and robust simulation tool that captures the key characteristic features of pathogenic proteins in Alzheimer’s disease by combining kinetic growth and fragmentation with network diffusion through a connectivity-weighted graph from the Human Connectome Project. Figure 1*c* suggests that—even with three orders of magnitude fewer degrees of freedom than the continuum models–our dynamic network model accurately predicts the typical spatio-temporal pattern of tau protein misfolding.

## 2. Kinetic models

To study the kinetics of protein misfolding, we consider three popular models with different levels of complexity, the simple one-concentration Fisher–Kolmogorov model [19], the two-concentration Heterodimer model [21] and the *n*-concentration Smoluchowski model [22].

### 2.1. The Fisher–Kolmogorov model

**Figure 2.**
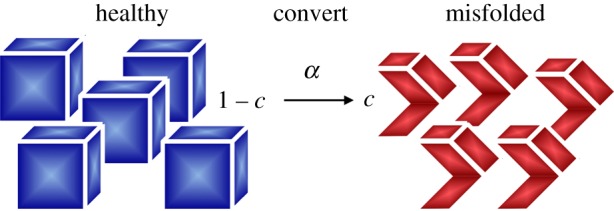
Kinetics of the Fisher–Kolmogorov model. The Fisher–Kolmogorov model has a single unknown, the misfolded protein concentration *c*. The model converts healthy to misfolded protein at a rate *α*. For the smallest perturbation from the healthy state, *c* > 0, all proteins will convert from the healthy to the misfolded state, *c* = 1. (Online version in colour.)

The simplest model to characterize protein misfolding is the Fisher–Kolmogorov model [19,20]. Initially proposed to model the spreading of a favoured gene in population dynamics, the Fisher–Kolmogorov model is now widely used to describe travelling wave solutions in ecology, physiology, combustion, crystallization, plasma physics, phase transition and biology [23]. It is based on a simple nonlinear reaction–diffusion equation for a single unknown, the misfolded protein concentration *c*,

2.1 $$\frac{d\;c}{d\;t}=\nabla \cdot (D\;\cdot \nabla c)+\alpha \;c\;[1-c],$$

where ***D*** is the diffusion tensor that characterizes global protein spreading and *α* characterizes the local conversion rate from the healthy to the misfolded state as illustrated in figure 2.

The Fisher–Kolmogorov equation (2.1) has two steady-state solutions, an unstable steady state at *c* = 0 and a stable steady state at *c* = 1. This implies that once misfolded protein is present anywhere in the brain, *c* > 0, the concentration will always be repelled from the benign state, *c* = 0, and attracted to the misfolded state, *c* = 1. While the Fisher–Kolmogorov model is attractive because of its simplicity and its low computational cost, its parameter *α* is purely phenomenological, it provides no insight into the mechanisms of infection, and it cannot capture intermediate equilibrium states as, for example, a result of pharmocological treatment.

### 2.2. The Heterodimer model

The simplest possible kinetic model that accounts for two configurations of the protein, the natural healthy state *p* and the misfolded state $\tilde{\;p}$, is the Heterodimer model [21]. In this model, misfolded proteins recruit healthy proteins at a rate *k*_11′_, healthy proteins bind to misfolded proteins and adopt their conformation at a rate *k*_1′2′_, and the resulting polymer fragments into infectious seeds at a rate *k*_2′2_,

2.2 $$p+\tilde{p}\overset{k_{11^{\prime }}}{\to }p\;\tilde{p}\;p\;\tilde{p}\overset{k_{1^{\prime }2^{\prime }}}{\to }\tilde{p}\;\tilde{p}\;\tilde{p}\;\tilde{p}\overset{k_{2^{\prime }2}}{\to }\tilde{p}+\tilde{p}.$$

For simplicity, we collectively represent the conformational conversion from the healthy to the misfolded state as a single step through the rate constant *k*_12_,

2.3 $$p+\tilde{p}\overset{k_{12}}{\to }\tilde{p}+\tilde{p}.$$

**Figure 3.**
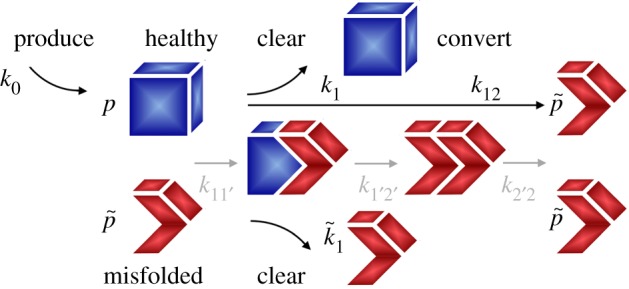
Kinetics of the Heterodimer model. The Heterodimer model has two unknowns, the healthy concentration *p* and the misfolded concentration $\tilde{p}$. The model produces healthy protein at a rate *k*_0_, clears healthy and misfolded protein at rates *k*_1_ and ${\tilde{k}}_{1}$, and converts healthy to misfolded protein at a rate *k*_12_, which collectively represents the processes of recruitment *k*_11′_, misfolding *k*_1′2′_ and fragmentation *k*_2′2_. (Online version in colour.)

These considerations motivate a system of governing equations for the spatio-temporal evolution of the total amount of healthy and misfolded proteins *p* and $\tilde{\;p}$ [24],

2.4 $$\begin{array}{rl} \frac{dp}{dt} & =\nabla \cdot (D\;\cdot \nabla p)+k_{0}-k_{1}\;p-k_{12}\;p\;\tilde{p}\\ \mathrm{and}\;\;\frac{d\;\tilde{p}}{d\;t} & =\nabla \cdot (D\;\cdot \nabla \tilde{p})-{\tilde{k}}_{1}\;\tilde{p}+k_{12}\;p\;\tilde{p}, \end{array}\}$$

where ***D*** is the diffusion tensor that characterizes protein spreading, *k*_0_ is the production rate of healthy protein, *k*_1_ and ${\tilde{k}}_{1}$ are the clearance rates of healthy and misfolded proteins, and *k*_12_ is the conversion rate from the healthy to the misfolded state, as illustrated in figure 3.

In the initial healthy state, the healthy and misfolded protein concentrations are *p*_0_ = *k*_0_/*k*_1_ and ${\tilde{p}}_{0}=0$; in the diseased state, they converge towards $p_{\infty }={\tilde{k}}_{1}/k_{12}$ and ${\tilde{p}}_{\infty }=k_{0}/{\tilde{k}}_{1}-k_{1}/k_{12}$.

We can simplify the Heterodimer model (2.4) by assuming that, initially, the amount of healthy protein is much larger than the amount of misfolded protein, $p\gg \tilde{p}$, which implies that d*p*/d*t* ≈ 0 and ∇⋅(D ⋅∇p)≈0. With these assumptions, equation (2.4) provides an explicit estimate of the amount of healthy protein *p*,

2.5 $$k_{0}-k_{1}\;p-k_{12}\;p\;\tilde{p}=0\;\text{thus}\;p=\frac{k_{0}}{k_{1}+k_{12}\;\tilde{p}}.$$

We approximate the healthy protein concentration *p* using a Taylor series, $p=k_{0}/k_{1}\;[1-\tilde{p}\;k_{12}/k_{1}]$, and substitute this expression into equation (2.4),

2.6 $$\frac{d\;\tilde{p}}{d\;t}=\nabla \cdot (D\;\cdot \nabla \tilde{p})+\left[k_{12}\frac{k_{0}}{k_{1}}-{\tilde{k}}_{1}\right]\;\tilde{p}-\frac{k_{12}^{2}\;k_{0}}{k_{1}^{2}}\;{\tilde{p}}^{2}.$$

By re-parametrizing equation (2.6) in terms of the misfolded protein concentration, $c=\tilde{p}/{\tilde{p}}^{\max}$ with ${\tilde{p}}^{\max}=k_{1}/k_{12}-k_{1}^{2}/k_{12}^{2}\;{\tilde{k}}_{1}/k_{0}$, we recover the special case of the Fisher–Kolmogorov model (2.1) for a single unknown, the misfolded protein concentration *c*,

2.7 $$\frac{d\;c}{d\;t}=\nabla \cdot (D\;\cdot \nabla c)+\alpha \;c\;[1-c]\;\text{with}\;\alpha =k_{12}\;\frac{k_{0}}{k_{1}}-{\tilde{k}}_{1}.$$

Interestingly, the Fisher–Kolmogorov parameter *α* now takes a physical interpretation in terms of the rates of production *k*_0_, clearance *k*_1_ and ${\tilde{k}}_{1}$, and conversion *k*_12_. While the Heterodimer model (2.4) strikes a natural balance between computational efficiency and mechanistic insight, it does not explicitly capture the size distribution of misfolded protein aggregates, their nucleation and fragmentation, and the response of the system to specific size-targeting antibodies.

### 2.3. The Smoluchowski model

To characterize the size distribution of misfolded protein aggregates, we consider the Smoluchowski model, a set of population balance equations that explicitly account for the kinetics of nucleation, aggregation, fragmentation and clearance of particles of different sizes [22]. For more than a century, the Smoluchowski model has been widely used in statistical physics to characterize processes of polymerization, coalescence of aerosols, emulsication and flocculation. It follows the *n* concentrations *c*_*i*_ of particles of size *i* = 1, …, *n* and explicitly models their aggregation and fragmentation through the individual aggregation and fragmentation rates *a*_*ij*_ and *f*_*ij*_ with *i*, *j* = 1, …, *n*,

2.8 $$c_{i}+c_{\;j}\to^{a_{ij}}\;c_{i+j}\;\text{and}\;c_{i+j}\overset{\;f_{ij}}{\to }c_{i}+c_{\;j}.$$

We can summarize the collective effects of aggregation and fragmentation on the concentration *c*_*i*_ through the aggregation and fragmentation *A*_*i*_ and *F*_*i*_,

2.9 $$\begin{array}{cc} & A_{i}=\overset{i-1}{\underset{\;j=1}{\sum }}a_{\;j(i-j)}c_{\;j}c_{i-j}-\overset{\infty }{\underset{\;j=1}{\sum }}2a_{ij}c_{i}c_{\;j}\\ \mathrm{and}\; & F_{i}=\overset{i-1}{\underset{\;j=1}{\sum }}f_{\;j(i-j)}c_{i}-\overset{\infty }{\underset{\;j=1}{\sum }}2f_{ij}c_{i+j}. \end{array}\}$$

Aggregation of two smaller particles *c*_*j*_ and *c*_*i*−*j*_ creates new particles *c*_*i*_ and removes particles *c*_*i*_ as they aggregate with *c*_*j*_ to larger particles *c*_*i*+*j*_. Fragmentation removes particles *c*_*i*_ as they fragment into two smaller particles *c*_*j*_ and *c*_*j*−*i*_ and adds new particles *c*_*i*_ from the fragmentation of larger particles *c*_*i*+*j*_ into *c*_*i*_ and *c*_*j*_. Taken together, the Smoluchowski model tracks the size distribution of particles through a nonlinear system of reaction–diffusion equations for the unknown concentrations *c*_*i*_ [25],

2.10 $$\frac{d\;c_{i}}{d\;t}=\nabla \cdot (D{\;}_{i}\cdot \nabla c_{i})+k_{0i}-k_{i}\;c_{i}+A_{i}-F_{i},$$

where ***D***_*i*_ is the size-specific diffusion tensor, *k*_0_ is the production rate, *k*_*i*_ is the clearance rate, and *A*_*i*_ and *F*_*i*_ are the size-specific aggregation and clearance rates according to aggregation–fragmentation kinetics (2.9). Here, we adopt a simplification of the Smoluchowski model (2.10), the nucleated polymerization model [26,27] with a nucleus size of two and spontaneous nucleation, to model the nucleation, aggregation, and fragmentation of tau proteins in Alzheimer’s disease [17] and make the following simplifying assumptions: we assume that diffusion is size-independent, ***D***_*i*_ = ***D***; production is only possible for healthy monomers, *k*_01_ = *k*_0_ for *i* = 1, but not for misfolded particles of any other size, *k*_0*i*_ = 0 for *i* > 1; clearance occurs at *k*_1_ for healthy monomers, is size-independent *k*_2_ = *k*_*i*_ for larger particles 1 < *i* < *n*; and impossible *k*_*n*_ = 0 for the largest particle size *i* = *n*; nucleation of two monomers occurs at a nucleation rate *a*_11_ = *κ* and is irreversible; aggregation of larger particles is size-independent *a*_*ij*_ = *a*, but can only occur by adding single monomers for *i* = 1 or *j* = 1 and is impossible *a*_*ij*_ = 0 otherwise; fragmentation into monomers is impossible *f*_*ij*_ = 0 for *i* = 1 or *j* = 1, fragmentation into larger particles is size-independent *f*_*ij*_ = *f* for 1 < *i*, *j* < *n*, and fragmentation is impossible *f*_*ij*_ = 0 for the largest particle size *i*, *j* = *n*. This results in the following explicit set of equations for the concentrations of sizes 1, 2, *i* = 3, …, *n* − 1, and *n* [28],

2.11  dc1dt=div(D⋅∇c1)+k0−k1c1−2κc12−ac1∑j=2n−1cj dc2dt=div(D⋅∇c2)−k2c2+κc12−ac1c2+2f∑j=2n−3 c2+j dcidt=div(D⋅∇ci)−ki ci−ac1ci+ac1ci−1+2f∑j=2n−i−1ci+j−f[i−3]cianddcndt=div(D⋅∇cn)+ac1cn−1.}

**Figure 4.**
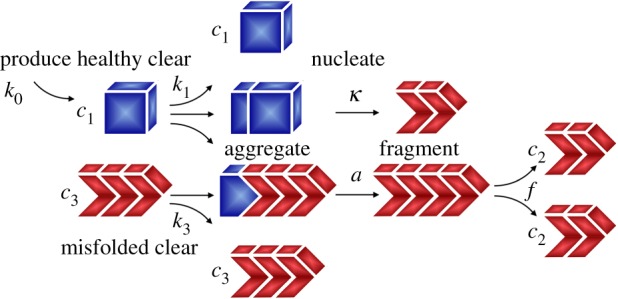
Kinetics of the Smoluchowski model. The Smoluchowski model has *n* unknowns *c*_*i*_, one for the concentration of each size *i* = 1, …, *n*, where *c*_1_ represents the concentration of healthy monomers. The model produces healthy protein at a rate *k*_0_, clears healthy and misfolded protein at rates *k*_1_ and *k*_*i*_, nucleates two misfolded proteins from two healthy proteins at a rate *κ*, aggregates by adding single healthy proteins to misfolded filaments at a rate *a*, and fragments misfolded filaments at a rate *f*. (Online version in colour.)

Figure 4 illustrates the Smoluchowski model for tau proteins. In addition to the global diffusion ***D***, this model has six local kinetic parameters, the production of healthy monomers *k*_0_, the clearance of healthy monomers *k*_1_ and misfolded polymers *k*_2_ = *k*_*i*_, the nucleation *κ*, the aggregation *a* and the fragmentation *f*. Notably, the Smoluchowski model features two distinct mechanisms to convert healthy proteins into misfolded proteins [17]: primary conversion reflected through the nucleation rate *κ* and secondary conversion reflected through the aggregation rate *a*. While the Smoluchowski model follows the size distribution of individual aggregates, allows for size-specific transport, aggregation, fragmentation and clearance, and provides a mechanistic interpretation of the protein misfolding [29], its intrinsic disadvantage is its large number of parameters and, with it, the risk of overfitting.

## 3. Brain network models

A defining feature of prion-like diseases is the spreading of misfolded proteins from a small infected region along axonal fibre tracts throughout the entire brain [30]. We model this spreading as the diffusion across the brain’s connectome [31], which we represent as a weighted undirected graph G with *N* nodes and *E* edges.

### 3.1. The connectivity-weighted graph

**Figure 5.**
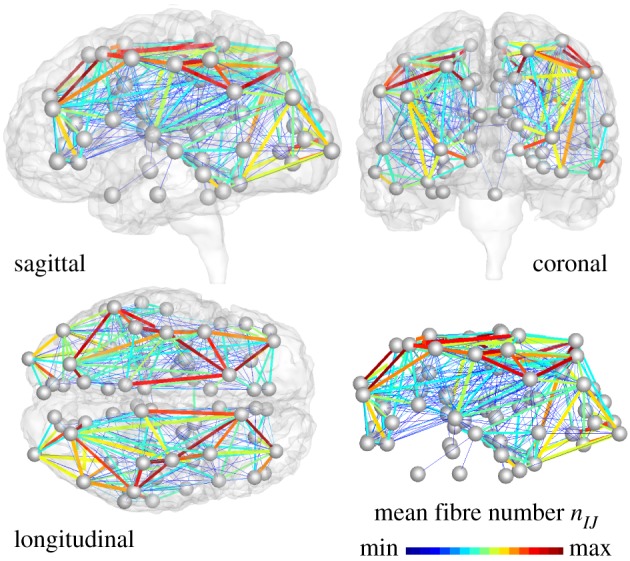
Brain network model. Misfolded tau proteins spread across the brain’s connectome represented as a weighted graph G with *N* = 83 nodes and *E* = 1130 edges. Edges are weighted by the mean fibre number *n*_*IJ*_ divided by the mean fibre length *l*_*IJ*_ averaged over 418 healthy brains from the Human Connectome Project. (Online version in colour.)

We extract the graph G from the tractography of diffusion tensor magnetic resonance images of 418 healthy subjects of the Human Connectome Project [32] using the Budapest Reference Connectome v. 3.0 [33]. While our method is generally applicable to graphs of any resolution, for illustrative purposes, we map the original graph with *N* = 1015 nodes and *E* = 37 477 edges onto a graph with *N* = 83 nodes and *E* = 1130 edges. This resolution corresponds to the widely used Freesurfer parcellation [34] and allows us to rapidly map the node representation back onto the brain surface. The degree of our initial graph, the number of edges per node, varies between 6 and 48, with fewest edges at the frontal pole and most edges at the caudate. We weight each edge by the mean fibre number *n*_*IJ*_ divided by the mean fibre length *l*_*IJ*_ averaged over all 418 brains. The mean fibre number varies between 1 ≤ *n*_*IJ*_ ≤ 596, with an average of ${\bar{n}}_{IJ}=40.2$ fibres per edge and most fibres between the superior parietal and the precuneus regions. The mean fibre length varies between 11.3 mm ≤ *l*_*IJ*_ ≤ 136.8 mm, with an average of ${\bar{l}}_{IJ}=38.40\;\text{mm}$ and the longest fibres between the lateral orbitofronal and the precuneus regions. Figure 5 illustrates our graph G with the edges colour-coded by the mean fibre number *n*_*IJ*_, mapped onto a three-dimensional brain model from magnetic resonance images [35].

### 3.2. The graph Laplacian

We summarize the connectivity of the graph G in terms of the degree matrix *D*_*II*_, a diagonal matrix that characterizes the degree of each node *I*, and the weighted adjacency matrix *A*_*IJ*_, the ratio of mean fibre number and length between nodes *I* and *J*. The difference between the degree matrix *D*_*IJ*_ and the adjacency matrix *A*_*IJ*_ defines the weighted graph Laplacian *L*_*IJ*_,

3.1 $$\begin{array}{rl} L_{IJ} & =D_{IJ}-A_{IJ}\;\mathrm{with}\;A_{IJ}=\frac{n_{IJ}}{l_{IJ}}\\ \mathrm{and}\;D_{II} & =\mathrm{diag}\overset{N}{\underset{J=1,J\neq I}{\sum }}A_{IJ}. \end{array}\}$$

**Figure 6.**
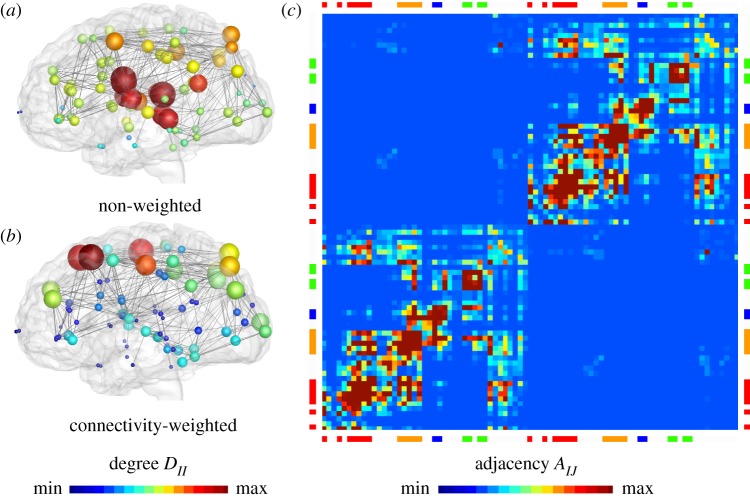
Brain network model. The connectivity of the graph G is represented through the degree *D*_*II*_, the number of edges per node, and the adjacency *A*_*IJ*_ = *n*_*IJ*_/*l*_*IJ*_, the ratio of fibre number and length. (*a*) Degree *D*_*II*_ of non-weighted and (*b*) connectivity-weighted graphs and (*c*) adjacency *A*_*IJ*_ of connectivity-weighted graph, averaged over 418 healthy brains from the Human Connectome Project. (Online version in colour.)

Figure 6*a* illustrates the degree *D*_*II*_ of the baseline non-weighted graph, and the degree *D*_*II*_ of our connectivity-weighted graph G (figure 6*b*) along with its adjacency *A*_*IJ*_ (figure 6*c*). For our connectivity-weighted graph, the degree varies between 2.1 ≤ *D*_*II*_ ≤ 127.6, with an average degree of ${\bar{D}}_{II}=42.8$ per node, and the lowest and highest degrees in the frontal pole, shown in blue, and in the precentral gyrus, shown in red. The adjacency matrix clearly reflects the small-world architecture of our brain with strongly connected hubs within the right and left hemispheres, indicated through the lower left and upper right quadrants, and strong connections within the four lobes, indicated through the eight red regions along the diagonal. The adjacency varies between 0.01 ≤ *A*_*IJ*_ ≤ 35.32, with an average adjacency of ${\bar{A}}_{IJ}=1.57$ per edge, and lowest and highest values between the superior parietal and the precuneus regions and between the lateral orbitofrontal and the isthmus cingulate regions. These pronounced variations in degree and adjacency confirm the general notion that the architecture of our brain resembles a small-world network [1] in which highly connected nodes are more likely to become infected and turn into hubs of misfolded protein spreading.

### 3.3. The network model

We assume that the weighted Laplacian *L*_*IJ*_ characterizes the spreading of healthy and misfolded proteins across the brain network and discretize our three kinetic models on our weighted undirected graph G. Specifically, we introduce the concentrations *c*_*I*_, *p*_*I*_, ${\tilde{p}}_{I}$ and *c*_*iI*_ as global unknowns at the *I* = 1, …, *N* nodes of our graph G. This results in the discretized sets of equations for the single concentration Fisher–Kolmogorov model (2.1) with *N* unknowns,

3.2 $$\frac{dc_{I}}{dt}=-\;\sum_{J=1}^{N}L_{IJ}c_{J}+\alpha c_{I}[1-c_{I}],\;$$

for two-concentration Heterodimer model (2.4) with 2 *N* unknowns,

3.3 $$\begin{array}{ccc} \; & \frac{d\;p_{I}}{dt}= & -\sum_{J=1}^{N}L_{IJ}\;p_{J}+k_{0}-k_{1}p_{I}-k_{12}p_{I}\;{\tilde{p}}_{I}\;\\ \mathrm{and}\; & \frac{d{\tilde{p}}_{I}}{dt}= & -\sum_{\;j=1}^{N}L_{IJ}{\tilde{p}}_{J}-{\tilde{k}}_{1}{\tilde{p}}_{I}+k_{12}\;p_{I}{\tilde{p}}_{I}\; \end{array}\}$$

and for the *n*-concentration Smoluchowski model (2.10) with *n* × *N* unknowns,

3.4 $$\frac{dc_{iI}}{dt}=-\;\sum_{J=1}^{N}L_{IJ}c_{iJ}+k_{0i}-k_{i}c_{iI}+A_{iI}-F_{iI}.$$

We discretize our network models in time using either implicit or explicit time integration schemes to simulate the spatio-temporal evolution of misfolded proteins across the brain.

## 4. Biomarker models

A biomarker is a global metric to characterize the evolution of neurodegeneration across the brain [36]. We calculate the biomarker abnormality as the temporal evolution of the total concentration of misfolded proteins integrated across a specific region of interest or across the brain as a whole. Biomarker abnormalities of the individual lobes provide insight into the spatio-temporal sequence of infection; global biomarker abnormalities of the brain as a whole provide a window into the progression of neurodegeneration and the time line of infection.

### 4.1. The Fisher–Kolmogorov model

**Figure 7.**
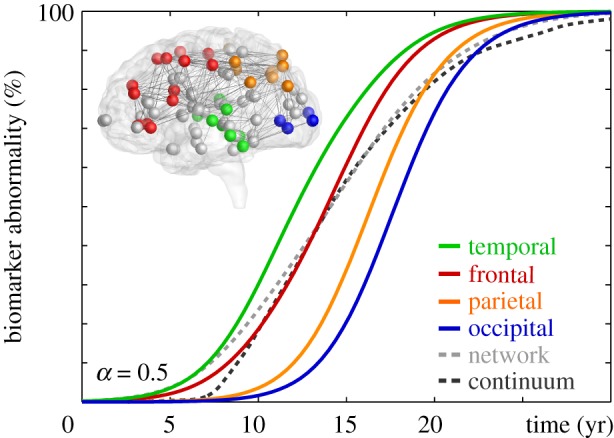
Biomarker abnormality of Fisher–Kolmogorov model. Integrating the concentration of misfolded proteins *c* across individual lobes reveals the characteristic activation sequence in Alzheimer’s disease from the temporal lobe to the frontal, parietal and occipital lobes. The dashed grey and black lines highlight the biomarker abnormality C of the Fisher–Kolmogorov network and continuum model in figure 1 integrated across the entire brain. (Online version in colour.)

Figure 7 summarizes the biomarker abnormality in all four lobes throughout a time period of three decades as predicted by the Fisher–Kolmogorov model (3.2). For the simulation, we chose a conversion rate constant of *α* = 0.5 and seeded misfolded proteins by increasing the initial concentration in the entorhinal cortex to *c*_0_ = 0.1. Our simulation uses an implicit time integration scheme with 100 times steps of Δ*t* = 0.4 years, and runs 0.55 and 0.64 s without and with output on a standard laptop computer. Figure 1*c* summarizes the resulting activation sequence. We post-process the simulation to calculate the biomarker abnormality,

4.1 $$C(t)=\;\overset{N}{\underset{I=1}{\sum }}c_{I}(t).$$

as the discrete sum of the misfolded protein concentration *c*_*I*_ at the *I* nodes of the temporal, frontal, parietal and occipital lobes, and of the brain as a whole. All biomarker curves in figure 7 display a smooth sigmoidal form, which is in excellent agreement with brain network spreading models in general [37] and with clinical biomarker models of neurodegeneration in particular [36]. The individual biomarkers of the four lobes reveal the characteristic spreading of misfolded tau protein in Alzheimer’s disease starting in the temporal lobe, shown in green, followed by the frontal, parietal, and occipital lobes, shown in red, orange, and blue. This activation sequence agrees well with the clinically observed spreading pattern [14] in figure 1*a*. For comparison, the dashed grey and black lines in figure 7 show the biomarker integrated over the entire brain as predicted by the continuum model [16] in figure 1*b* and by the Fisher–Kolmogorov network model in figure 1*c*. This quantitative comparison confirms that, even at a much lower spatial resolution, our network model captures the integral characteristics of continuum models for Alzheimer’s disesase [38].

### 4.2. The Heterodimer model

**Figure 8.**
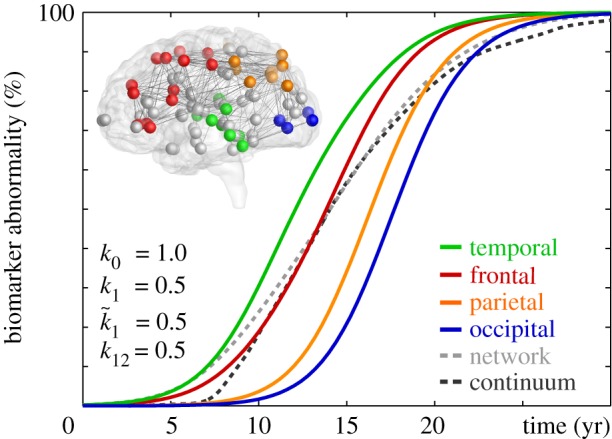
Biomarker abnormality of Heterodimer model. Integrating the concentration of misfolded proteins $\tilde{p}$ across individual lobes reveals the characteristic activation sequence in Alzheimer’s disease from the temporal lobe to the frontal, parietal and occipital lobes. The dashed grey and black lines highlight the biomarker abnormality $\tilde{P}$ of the Heterodimer network and continuum model in figure 1 integrated across the entire brain. (Online version in colour.)

Figure 8 summarizes the biomarker abnormality in all four lobes, similar to figure 7, but now, instead of using the one-concentration Fisher–Kolmogorov model (3.2), using the two-concentration Heterodimer model (3.3). For the simulation, we chose a production rate of *k*_0_ = 1.0, clearance rates of *k*_1_ = 0.5 and ${\tilde{k}}_{1}=0.5$, and a conversion rate of *k*_12_ = 0.5, which results in an initial healthy concentration of *p*_0_ = 2.0 and an initial misfolded concentration of ${\tilde{p}}_{0}=0.0$, which we increased locally in the entorhinal cortex to ${\tilde{p}}_{0}=0.1$ to seed misfolding. Our simulation uses an implicit time integration scheme with 100 times steps of Δ*t* = 0.4 years, and runs slightly longer than the Fisher–Kolmogorov model, but still completes in 0.66 and 0.68 s without and with output on a standard laptop computer. Again we calculate the biomarker abnormality,

4.2 $$\tilde{P}(t)=\;\overset{N}{\underset{I=1}{\sum }}{\tilde{p}}_{I}(t).$$

as the discrete sum of the misfolded protein concentration ${\tilde{p}}_{I}$ at the *I* nodes of the four lobes and of the brain as a whole. Figure 8 confirms that, for a conversion rate, $\alpha =k_{12}\;k_{0}/k_{1}-{\tilde{k}}_{1}$ in accordance with equation (2.7), the Heterodimer model predicts an identical infection sequence and similar biomarker curves as the Fisher–Kolmogorov model: The green, red, orange and blue curves of the individual lobes and the dashed grey curve of the whole brain in figure 8 are indistinguishable from the curves in figure 7. This suggests that, if we are exclusively interested in the spreading of misfolded protein, from an initial healthy to a fully misfolded state, we can use the simple Fisher–Kolmogorov model (3.2) without loss of accuracy and interpret its phenomenological rate constant *α* as a combination of the mechanistic rate constants *k*_0_, *k*_1_, ${\tilde{k}}_{1}$ and *k*_12_ of the Heterodimer model.

### 4.3. Infection times

**Figure 9.**
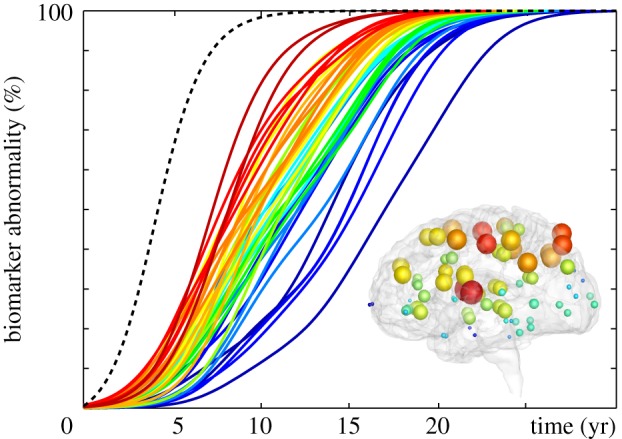
Infection times. Biomarker curves for misfolded protein seeding in *N* = 83 seeding regions illustrate the regional vulnerability of the network model. Misfolded proteins spread fastest when seeded in the putamen or insula, red spheres and curves, and slowest when seeded in the frontal pole and entorhinal region, blue spheres and curves. The dashed grey line highlights the lower limit of the infection time associated with a homogeneous seeding across all regions. (Online version in colour.)

We now adopt the Heterodimer model to study the regional vulnerability of different brain regions. Specifically, we successively seed misfolded protein in all *N* = 83 regions, simulate the spatio-temporal spreading across the brain, calculate the resulting 83 biomarker curves, and quantify the individual infection times. Figure 9 summarizes the biomarker curves and their associated brain regions colour-coded by infection time. Misfolded proteins spread fastest when seeded in the putamen or insula with a total infection times of 20.2 years, shown in red, and slowest when seeded in the frontal pole and entorhinal region with infection times of 30.4 and 28.8 years, shown in blue. The significant variation in infection times, by more than 10 years, underlines the heterogeneity of the brain network with a few highly connected hubs [1]. These observations agree well with the hierarchical spread of epidemic outbreaks known from general network theory [39]. For comparison, the dashed grey line illustrates the lower limit of the infection time of 16.6 years, associated with a homogeneous seeding across all *N* = 83 regions. The mean infection time of 24.9 years on the heterogeneous network is almost exactly 50% longer. Interestingly, the entorhinal cortex, which is known as the region where misfolded tau proteins are first observed [14], is associated with the second longest in infection time. This could explain, at least in part, why tau pathology is so difficult to detect during the early stages of Alzeimer’s disease [13]. The heterogeneous vulnerability of the brain network in figure 9 presents opportunities when designing treatment strategies: Reducing the local accumulation of misfolded protein in highly infectious regions such as the putamen or the insula will have a more pronounced effect on slowing down neurodegeneration than intervening in poorly connected regions such as the frontal pole. The following section focusses on different potential treatment options.

## 5. Treatment opportunities

Now that we have established a solid baseline simulation for the progression of Alzheimer’s disease that agrees well with clinical observations, we explore the potential of our models for simulating different treat opportunities. Two promising therapeutic strategies are currently emerging to delay or even prevent the progression of Alzheimer’s disease [40]: reducing misfolding [41] and increasing clearance [42]. Even if, to date, we do not have a precise knowledge about all the model parameters, we can still perform numerical experiments to elaborate the mechanisms and time scales associated with these interventions.

### 5.1. Delaying conversion

**Figure 10.**
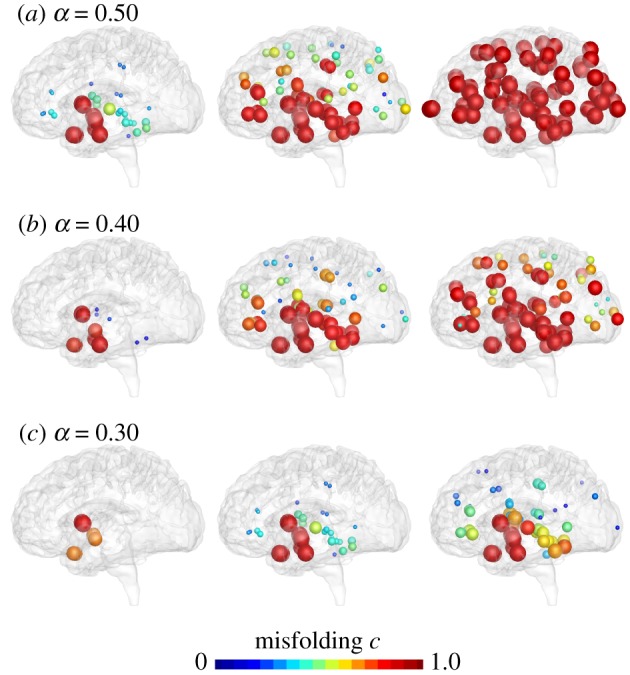
Delaying conversion. The Fisher–Kolmogorov model predicts that lower conversion rates *α* delay the increase of misfolded protein *c*. Baseline Alzheimer’s disease (*a*) and Alzheimer’s disease with moderately (*b*) and markedly (*c*) reduced conversion *α* from the healthy to the misfolded state. (Online version in colour.)

**Figure 11.**
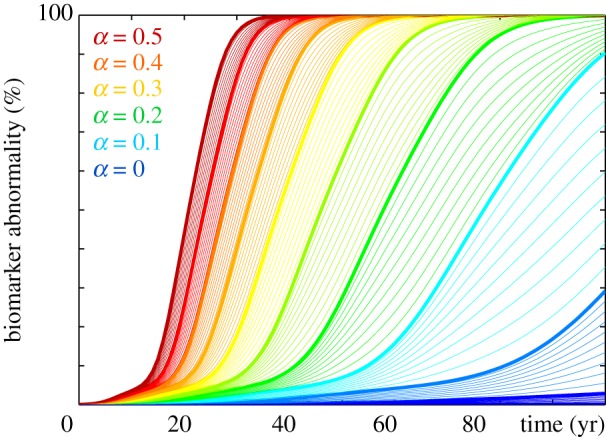
Reducing biomarker abnormality through delayed conversion. Decreasing the conversion *α* delays the accumulation of the misfolded protein concentration *c* and with it the biomarker abnormality C. Irrespective of the conversion rate *α*, the misfolded protein concentration of the Fisher–Kolmogorov model always converges towards the fully misfolded state with a biomarker abnormality of C=100%. (Online version in colour.)

Figures 10 and 11 reveal the effects of reducing misfolding and increasing clearance with the Fisher–Kolmogorov model. The only model parameter is the conversion rate $\alpha =k_{12}\;k_{0}/k_{1}-{\tilde{k}}_{1}$, which we can interpret as a combination of production *k*_0_, clearance *k*_1_ and ${\tilde{k}}_{1}$, and conversion *k*_12_. Figures 10 and 11 illustrate simulations of the baseline case with *α* = 0.5, and reduced values of *α*, which collectively mimic reduced misfolding *k*_12_ and increased clearance ${\tilde{k}}_{1}$. The simulations confirm our intuition that decreasing the conversion *α* delays the accumulation of the misfolded protein *c* and with it the biomarker abnormality C. However, figures 10 and 11 also illustrate an inherent limitation of the Fisher–Kolmogorov model: once misfolded protein is present anywhere in the brain, the concentration will always be repelled from the healthy state and attracted to the misfolded state. While the Fisher–Kolmogorov model (2.1) is a simple model to efficiently explore the dynamics of protein misfolding on more complex three-dimensional finite-element geometries [16] and to study the interplay of biochemical and biomechanical degeneration [38], for applications with intermediate states, we recommend using more mechanistic kinetic models like the Heterodimer model (2.4) or the Smoluchowski model (2.10).

### 5.2. Reducing misfolding

**Figure 12.**
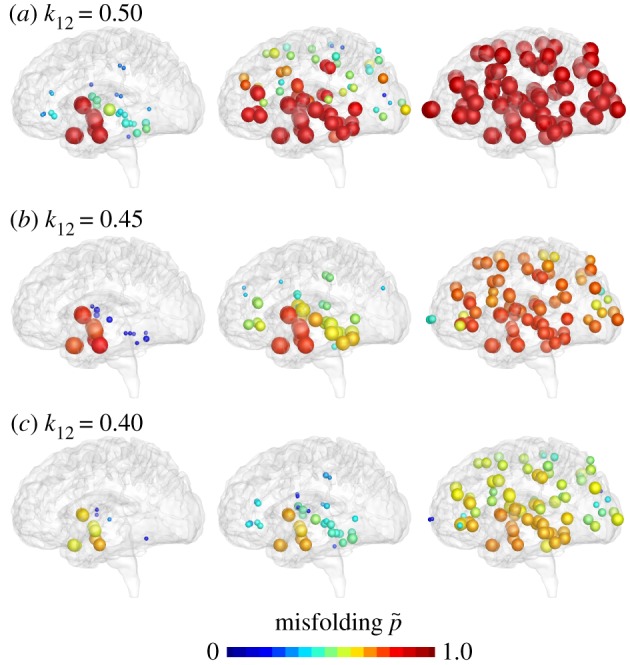
Reducing misfolding. The Heterodimer model predicts that lower turnover rates *k*_12_ delay and reduce the accumulation of misfolded protein $\tilde{p}$. Baseline Alzheimer’s disease (*a*) and Alzheimer’s disease with moderately (*b*) and markedly (*c*) reduced turnover *k*_12_ from the healthy to the misfolded state. (Online version in colour.)

**Figure 13.**
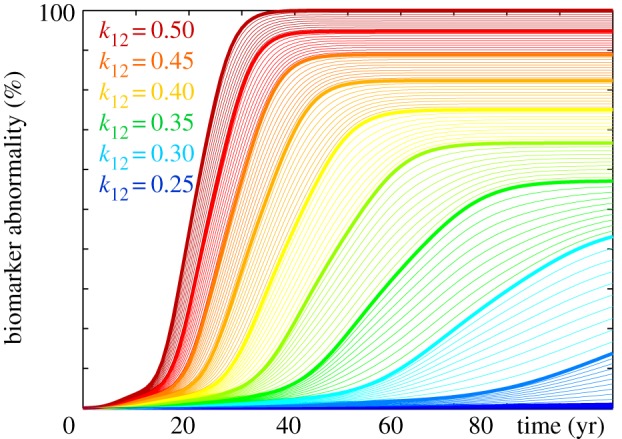
Reducing biomarker abnormality through reduced misfolding. Decreasing the turnover *k*_12_ delays and reduces the accumulation of misfolded tau protein $\tilde{p}$ and with it the biomarker abnormality $\tilde{P}$. Depending on the turnover rate *k*_12_, the misfolded protein concentration of the Heterodimer model can converge towards intermediate states with a reduced biomarker abnormality of $\tilde{P}<100\text{\%}$. (Online version in colour.)

Figures 12 and 13 illustrate the effect of reducing misfolding with the Heterodimer model. A turnover rate of *k*_12_ = 0.50 predicts the baseline progression of Alzheimer’s disease in agreement with figure 1. For this baseline case with a production of *k*_0_ = 1.0, clearances of *k*_1_ = 0.5 and ${\tilde{k}}_{1}=0.5$, and a conversion of *k*_12_ = 0.5 the Heterodimer model is identical to the Fisher–Kolmogorov model with a conversion of *α* = 0.50. According to equation (2.7), decreasing the Heterodimer conversion to *k*_12_ = 0.40 would correspond to decreasing the Fisher–Kolmogorov conversion to *α* = 0.3. However, the *k*_12_ = 0.40 pattern in figure 12 and the *α* = 0.3 pattern in figure 10 show significant differences: decreasing the turnover rate moderately to *k*_12_ = 0.45 and markedly to *k*_12_ = 0.40 not only delays but also reduces the accumulation of misfolded protein $\tilde{p}$ and with it the biomarker abnormality $\tilde{P}$. Strikingly, in the early stages of neurodegeneration, even a small reduction of misfolding can delay disease progression by several decades [41] and reduce the resting state of misfolded protein ${\tilde{p}}_{\infty }=k_{0}/{\tilde{k}}_{1}-k_{1}/k_{12}$ below its untreated value, here to ${\tilde{p}}_{\infty }=0.89$ for *k*_12_ = 0.45 and to ${\tilde{p}}_{\infty }=0.75$ for *k*_12_ = 0.40.

### 5.3. Increasing clearance

**Figure 14.**
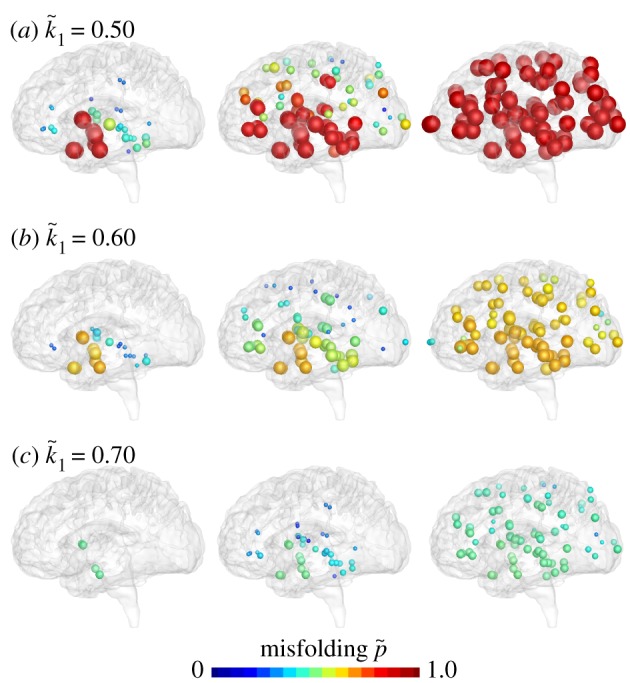
Increasing clearance. The Heterodimer model predicts that higher clearance rates ${\tilde{k}}_{1}$ delay and reduce the accumulation of misfolded protein $\tilde{p}$. Baseline Alzheimer’s disease (*a*) and Alzheimer’s disease with moderately (*b*) and markedly (*c*) increased clearance ${\tilde{k}}_{1}$ of misfolded protein. (Online version in colour.)

**Figure 15.**
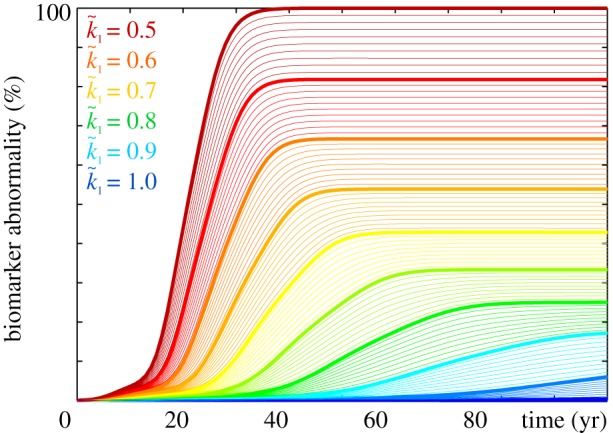
Reducing biomarker abnormality through increased clearance. Increasing the clearance ${\tilde{k}}_{1}$ delays and reduces the accumulation of misfolded tau protein $\tilde{p}$ and with it the biomarker abnormality $\tilde{P}$. Depending on the clearance rate ${\tilde{k}}_{1}$, the misfolded protein concentration of the Heterodimer model can converge towards intermediate states with a reduced biomarker abnormality of $\tilde{P}<100\text{\%}$. (Online version in colour.)

Figures 14 and 15 highlight the effect of increasing clearance with the Heterodimer model. A clearance rate of ${\tilde{k}}_{1}=0.50$ predicts the baseline progression of Alzheimer’s in agreement with figure 1. For this baseline case, the Heterodimer model is identical to the Fisher–Kolmogorov model with a conversion of *α* = 0.50. According to equation (2.7), increasing the clearance to ${\tilde{k}}_{1}=0.70$ would correspond to decreasing the conversion to *α* = 0.3. But, similar to the previous example, the ${\tilde{k}}_{1}=0.70$ pattern in figure 14 and the *α* = 0.3 pattern in figure 10 show significant differences: increasing the clearance rate ${\tilde{k}}_{1}$ has similar effects as decreasing the turnover rate *k*_12_; it not only delays but also reduces the accumulation of misfolded protein $\tilde{p}$ and with it the biomarker abnormality P. Similar to a decreased turnover, an increased clearance can delay disease progression by several decades [42] and reduce the resting state of misfolded protein ${\tilde{p}}_{\infty }=k_{0}/{\tilde{k}}_{1}-k_{1}/k_{12}$ significantly below its untreated value, here to ${\tilde{p}}_{\infty }=0.67$ for ${\tilde{k}}_{1}=0.60$ and to ${\tilde{p}}_{\infty }=0.43$ for ${\tilde{k}}_{1}=0.70$. While the Heterodimer model provides valuable insight into the clearance of all misfolded proteins, it cannot predict the effect of the selective clearance of small molecules.

## 6. Size matters

In this last example, we explore the interplay of nucleation, aggregation, fragmentation and the general distribution of particle size using the Smoluchowski model and highlight its advantages over the Fisher–Kolmogorov and Heterodimer models.

### 6.1. Biomarker spectrum

**Figure 16.**
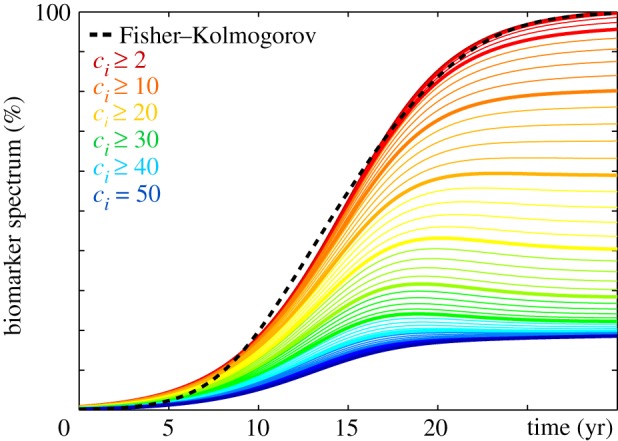
Biomarker spectrum of Smoluchowski model. Integrating the mass of misfolded proteins *i* *c*_*i*_ larger than a critical toxic size *i* reveals the characteristic nucleation, aggregation, fragmentation dynamics in Alzheimer’s disease. The red line $M_{2}$ indicates the total mass of all misfolded proteins *c*_*i*_ ≥ 2; the blue line $M_{50}$ indicates the mass of all aggregates of the largest size *c*_*i*_ = 50. The dashed black line highlights the biomarker abnormality C of the Fisher–Kolmogorov model in figure 7 and $\tilde{P}$ of the two-concentration model in figure 8. (Online version in colour.)

To illustrate the dynamics of the Smoluchowski model, we consider the biomarker spectrum of the principal moments of the size distribution, the overall concentration of misfolded proteins *c*_*i*_ above a critical size *i* ≥ *j*,

6.1 $$C_{\;j}(t)=\sum_{I=1}^{N}\sum_{i=j}^{n}\;c_{iI}(t),$$

and the overall mass of misfolded proteins *i* *c*_*i*_ above a critical size *i* ≥ *j*,

6.2 $$M_{\;j}(t)=\sum_{I=1}^{N}\sum_{i=j}^{n}\;i\;c_{iI}(t),$$

where *c*_*iI*_ denotes the concentration of proteins of length *i* = 1, …, *n* at node *I* = 1, …, *N*. Specifically, $C_{2}$ and $M_{2}$ denote the total aggregate concentration and the total aggregate mass, and $\bar{c}=M_{2}/C_{2}$ denotes the mean aggregate length. Figure 16 illustrates the biomarker spectrum $M_{\;j}$ for a simulation with a production rate of *k*_0_ = 1.0, clearance rates of *k*_1_ = 0.5 for healthy monomers, *k*_2_ = *k*_*i*_ = 0.5 for aggregates, and *k*_*n*_ = 0.0 for the particles of the largest size, an aggregation rate of *a* = 10.0, a fragmentation rate of *f* = 0.048, and nucleation rates of *κ* = 0.00016 in the entorhinal cortex and *κ* = 0.0 in all other regions. There is a large uncertainty about the true values for these rate constants and they may differ highly between variants [26]. Naturally, the choice of these rates will affect the sequence of events and the interplay of nucleation, aggregation, fragmentation and spread [28]. Here, we chose the parameter values such that their order of magnitude closely followed reported values in the literature [43], where the largest rate constant is the monomer production *k*_0_ followed by the monomer clearance *k*_1_, the polymer clearance *k*_*i*_, the fragmentation *f* and the nucleation *κ*. Because of the small time constants, we now have to use a finer time discretization with 1000 times steps of Δ*t* = 0.04 years, and we now use an explicit time integration. Our average simulation with *n* = 50 discrete aggregate sizes runs 1.97 and 2.20 s without and with output on a standard laptop computer; increasing the number of aggregates to *n* = 500 increases the simulation time to 6.96 and 16.43 seconds without and with output. Interestingly, the mass of all misfolded proteins $M_{2}$, the dark red curve in figure 16, is almost indistinguishable from dashed black curve that highlights the biomarkers C of the Fisher–Kolmogorov model in figure 7 and $\tilde{P}$ of the Heterodimer model in figure 8. However, in contrast to the Fisher–Kolmogorov and Heterodimer models, the Smoluchowski model features several competing mechanisms and time scales and allows for two distinct types of conversion, nucleation and aggregation. To better understand the dynamics of the Smoluchowski model, we explored the parameter space and learned that increasing the production *k*_0_, the aggregation *a*, or the fragmentation *f* accelerates and increases protein misfolding and shifts the curves in figure 16 to the left and upward; increasing the nucleation *κ* accelerates protein misfolding and shifts the curves to the left, but not upward; and increasing the clearance *k*_1_ or *k*_2_ = *k*_*i*_ decelerates and reduces protein misfolding and shifts the curves to the right and downward. The initial time delay between the red $M_{2}$ curve for *c*_*i*_ ≥ 2 and the blue $M_{50}$ curve for *c*_*i*_ = 50 illustrates the aggregation dynamics, and confirms our intuition that smaller particles have to form first to trigger the aggregation of larger particles. Intermediate aggregate sizes, 20 ≤ *c*_*i*_ ≤ 30, highlighted through the yellow $M_{20}$ to green $M_{30}$ curves, display a small bump and increase initially, but then either partially clear or fragment into smaller aggregates.

### 6.2. Aggregate size distribution

**Figure 17.**
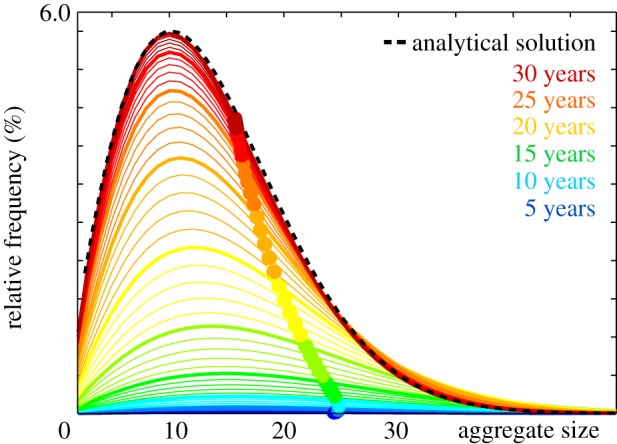
Emerging aggregate size distribution. During the early stages of infection, the mean aggregate size increases; during the later stages, it decreases gradually towards a homeostatic mean. Colour-coded lines indicate the evolution of aggregate size and frequency; dots show the evolution of the mean aggregate size; dashed black line highlights the analytical solution [43]. (Online version in colour.)

Figure 17 illustrates the emerging aggregate size distribution and explains the dynamic features of the model: strikingly, the mean particle size, highlighted through the colour-coded dots, increases during the early stages of infection up to a mean particle size of 24.8 after 5.3 years, but then decreases gradually during the later stages towards a converged mean particle size of 15.7 after 30 years. This agrees well with the dynamics of the Smoluchowski model, which is known to predict an initial increase of the average particle size followed by a gradual decrease towards the homeostatic mean [43]. The red curve of the converged aggregate size distribution agrees well with the dashed black line of the analytical solution [43].

**Figure 18.**
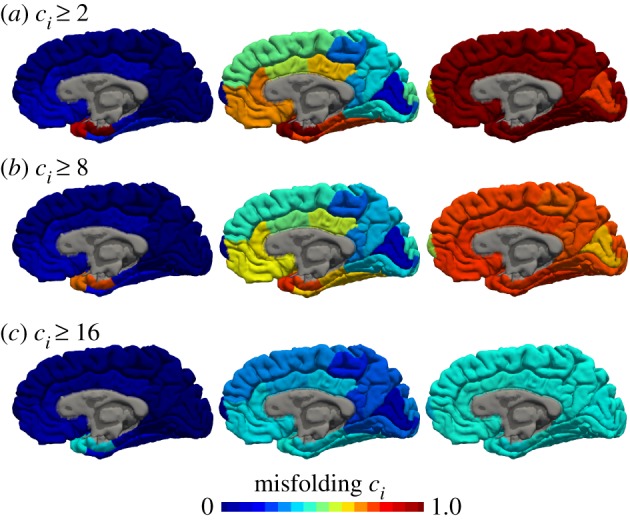
Typical pattern of tau aggregation in Alzheimer’s disease. The Smoluchowski model predicts that misfolded tau proteins occur first in the locus coeruleus and transentorhinal layer from where they spread to the transentorhinal region and the proper entorhinal cortex and ultimately affect all interconnected neocortical brain regions. Smaller aggregates emerge first (*a*) and grow progressively into moderate (*b*) and large (*c*) aggregates. (Online version in colour.)

Figure 18 illustrates the spatio-temporal evolution of aggregates of different sizes. For illustrative purposes, rather then showing the discrete network with the individual *N* = 83 nodes, we colour-coded the associated 83 brain regions according to their misfolded protein concentration using the software tool Freesurfer [34]. The time lapse images show that misfolded tau proteins occur first in the locus coeruleus and transentorhinal layer from where they spread to the transentorhinal region and the proper entorhinal cortex and ultimately affect all interconnected neocortical brain regions. This spreading pattern agrees well with clinical observations [15], the continuum model of tau protein spreading [16], and our predictions with the Fisher–Kolmogorov and Heterodimer models in figure 1. Initially, the emerging aggregates are small, but they grow into progressively larger sizes as suggested by the biomarker spectrum in figure 16, and converge towards the final aggregate size distribution as indicated in figure 17. While analytical approximations exist to predict the biomarker curve and final size distribution for size-independent model parameters [43], numerical methods are necessary to predict the effect of selective parameter changes on these global readouts of the model.

### 6.3. Size-specific treatment

**Figure 19.**
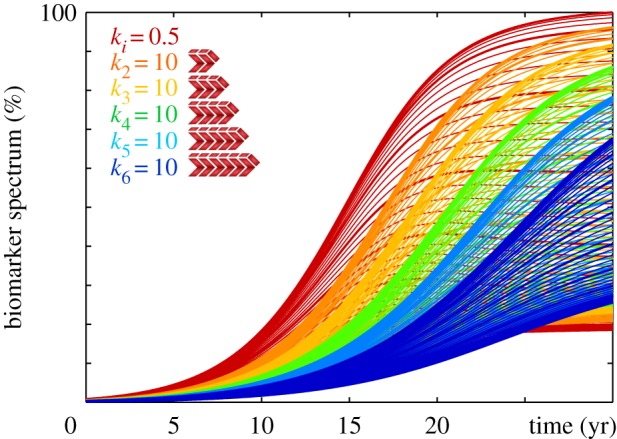
Reducing biomarker spectrum through size-targeted clearance. Increasing the clearance *k*_*i*_ of a single specific aggregate size delays and reduces the accumulation of misfolded tau protein *c*_*i*_ and with it the biomarker spectrum M. Increasing the target size of clearance, here from *c*_2_ to *c*_6_ decelerates and reduces protein misfolding and narrows the overall size distribution. The red curves highlight the biomarker spectrum for the baseline model with a homogeneous clearance in figure 16. (Online version in colour.)

Figure 19 highlights the effect of selective size-targeted clearance on the biomarker spectrum as predicted by the numerical Smoluchowski model. A homogeneous clearance rate of *k*_*i*_ = 0.50 as indicated by the red biomarker spectrum, predicts the baseline progression of Alzheimer’s disease in agreement with figure 16. Increasing the clearance of a single specific aggregate size *i* from *k*_*i*_ = 0.5 to *k*_*i*_ = 10, while keeping all other clearance rates unchanged, delays and reduces the accumulation of misfolded tau protein *c*_*i*_ and with it the biomarker spectrum M. Clearing a specific aggregate size *i* at a higher rate not only affects smaller aggregate sizes through a reduced fragmentation but also larger aggregate sizes through a reduced aggregation, which, collectively, results in an overall narrower biomarker spectrum. Increasing the target size of clearance, here from *c*_2_ shown in orange to *c*_6_ shown in blue decelerates and reduces protein misfolding and increases narrowing of the overall spectrum. Taken together, while the Fisher–Kolmogorov model and the Heterodimer model provide valuable insight into the kinetics of protein misfolding, only the Smoluchowski model can explain the interplay between primary conversion through nucleation, secondary conversion through aggregation, and the general distribution of particle size. Although targeting a single specific size might seem rather hypothetical, we can envision therapeutic approaches that target the production or clearance of small particles below a characteristic size [44].

## 7. Conclusion

Despite their complexity, neurodegenerative diseases display remarkably consistent histopathological patterns. In Alzheimer’s disease, these invasion patterns are highly correlated with the spreading of misfolded amyloid beta and tau proteins. Here, we modelled the spreading of tau proteins by combining misfolding kinetics and network diffusion through a connectivity-weighted graph. In our dynamic brain network model, the concentrations of healthy and misfolded protein emerge dynamically at each node and propagate across the graph through its connectivity-weighted edges. Our model correctly predicts the spatio-temporal spreading pattern of tau in Alzheimer’s disease. There is currently no *in vivo* technology to quantify these spreading patterns longitudinally and non-invasively in humans. Our model provides a computational window into the interacting time scales and mechanisms of neurodegeneration. Its computational efficiency allows us to rapidly screen the landscape of disease-specific parameters that govern the kinetics of protein misfolding and spreading. We have demonstrated the potential of our model by simulating biomarker curves, aggregate size distributions, infection times and therapeutic intervention. A better understanding of the spreading of misfolded proteins could open new therapeutic opportunities towards blocking protein misfolding and promoting protein clearance using antibodies or small molecules. Ultimately, we envision that brain network models can help us answer some of the fundamental open questions in neurodegeneration: Why do neurodegenerative diseases progress so slowly, but yet so highly reproducibly? Can we identify early biomarkers of neurodegeneration that would allow us to interfere early? Why is neurodegeneration currently unstoppable? Can we interfere therapeutically and what would be the best time to do so? What are the roles of intra- and extracellular propagation? Can we manipulate spreading and where do we best interfere? What is the timeline of neurodegeneration? Can we predict personalized risk curves for individuals and estimate the socio-economic burden for an entire population? While we are still far from answering these questions, we believe that quantitative brain network modelling is a promising step towards identifying the key mechanisms of neurodegeneration and their roles in neurodegenerative disease.

## Data accessibility

This article does not contain any additional data.

## References

[1] Bassett DS, Bullmore E 2006 Small-world brain networks. Neuroscientist 12, 512–523. (10.1177/1073858406293182) 17079517

[2] Bassett DS, Bullmore ET 2017 Small-world brain networks revisited. Neuroscientist 23, 499–516. (10.1177/1073858416667720) 27655008 PMC5603984

[3] Bullmore E, Sporns O 2009 Complex brain networks: graph theoretical analysis of structural and functional systems. Nat. Rev. Neurosci. 10, 186–198. (10.1038/nrn2575) 19190637

[4] Kuhl E 2019 Connectomics of neurodegeneration. Nat. Neurosci. 22, 1200–1202. (10.1038/s41593-019-0459-3) 31346294

[5] Raj A, Kuceyeski A, Weiner M 2012 A network diffusion model of disease progression in dementia. Neuron 73, 1204–1215. (10.1016/j.neuron.2011.12.040) 22445347 PMC3623298

[6] Iturria-Medina Y, Sotero RC, Toussaint PJ, Evans AC, Initiative ADN 2014 Epidemic spreading model to characterize misfolded proteins propagation in aging and associated neurodegenerative disorders. PLoS Comput. Biol. 10, e1003956 (10.1371/journal.pcbi.1003956) 25412207 PMC4238950

[7] Jucker M, Walker LC 2011 Pathogenic protein seeding in Alzheimer disease and other neurodegenerative disorders. Ann. Neurol. 70, 532–540. (10.1002/ana.v70.4) 22028219 PMC3203752

[8] Prusiner SB 1998 Prions. Proc. Natl Acad. Sci. USA 95, 13 363–13 383. (10.1073/pnas.95.23.13363) 9811807 PMC33918

[9] Ittner LM, Gotz J 2011 Amyloid-beta and tau – a toxic pas de deux in Alzheimer’s disease. Nat. Rev. Neurosci. 12, 67–72. (10.1038/nrn2967) 21193853

[10] Goedert M 2015 Alzheimer’s and Parkinson’s diseases: the prion concept in relation to assembled A*β*, tau, and *α*-synuclein. Science 349, 1255555 (10.1126/science.1255555) 26250687

[11] Eisenberg D, Jucker M 2012 The amyloid state of proteins in human diseases. Cell 148, 1188–1203. (10.1016/j.cell.2012.02.022) 22424229 PMC3353745

[12] Bressloff PC, Newby JM 2013 Stochastic models of intracellular transport. Rev. Mod. Phys. 85, 135–196. (10.1103/RevModPhys.85.135)

[13] Walker LC, Jucker M 2015 Neurodegenerative diseases: expanding the prion concept. Annu. Rev. Neurosci. 38, 87–103. (10.1146/annurev-neuro-071714-033828) 25840008 PMC4803040

[14] Braak H, Braak E 1991 Neuropathological stageing of Alzheimer-related changes. Acta Neuropathol. 82, 239–259. (10.1007/BF00308809) 1759558

[15] Jucker M, Walker LC 2013 Self-propagation of pathogenic protein aggregates in neurodegenerative diseases. Nature 501, 45–51. (10.1038/nature12481) 24005412 PMC3963807

[16] Weickenmeier J, Kuhl E, Goriely A 2018 The multiphysics of prion-like diseases: progression and atrophy. Phys. Rev. Lett. 121, 158101 (10.1103/PhysRevLett.121.158101) 30362787

[17] Knowles TPJ, Waudby CA, Devlin GL, Cohen SIA, Aguzzi A, Vendruscolo M, Terentjev EM, Welland ME, Dobson CM 2009 An analytical solution to the kinetics of breakable filament assembly. Science 326, 1533–1537. (10.1126/science.1178250) 20007899

[18] Weickenmeier J, Jucker M, Goriely A, Kuhl E 2019 A physics-based model explains the prion-like features of neurodegeneration in Alzheimer’s disease, Parkinson’s disease, and amyotrophic lateral sclerosis. J. Mech. Phys. Solids 124, 264–281. (10.1016/j.jmps.2018.10.013)

[19] Fisher RA 1937 The wave of advance of advantageous genes. Ann. Eugen. 7, 355–369. (10.1111/j.1469-1809.1937.tb02153.x)

[20] Kolmogorov AN 1937 A study of the equation of diffusion with increase in the quantity of matter, and its application to a biological problem. Moscow Univ. Bull. Math. 1, 1–25.

[21] Prusiner SB *et al.* 1990 Transgenic studies implicate interactions between homologous PrP isoforms in scrapie prion replication. Cell 63, 673–686. (10.1016/0092-8674(90)90134-Z) 1977523

[22] Smoluchowski M 1916 Drei Vorträge über diffusion, Brownsche Molekularbewegung, und Koagulation von Kollidteilchen. Phys. Z. 17, 557–5171, 585–599.

[23] Simpson MJ, Treloar KK, Binder BJ, Haridas P, Manton KJ, Leavesley DI, McElwain DLS, Baker RE 2013 Quantifying the role of cell motility and cell proliferation in a circular barrier assay. J. R. Soc. Interface 10, 20130007 (10.1098/rsif.2013.0007) 23427098 PMC3627085

[24] Matthäus F 2006 Diffusion versus network models as descriptions for the spread of prion diseases in the brain. J. Theor. Biol. 240, 104–113. (10.1016/j.jtbi.2005.08.030) 16219329

[25] Bertsch M, Franchi B, Marcello N, Tesi MC, Tosin A 2017 Alzheimer’s disease: a mathematical model for onset and progression. Math. Med. Biol. 34, 193–214. (10.1093/imammb/dqw003) 27079222

[26] Masel J, Jansen VA, Nowak MA 1999 Quantifying the kinetic parameters of prion replication. Biophys. Chem. 77, 139–152. (10.1016/S0301-4622(99)00016-2) 10326247

[27] Greer ML, Pujo-Menjouet L, Webb GF 2006 A mathematical analysis of the dynamics of prion proliferation. J. Theor. Biol. 242, 598–606. (10.1016/j.jtbi.2006.04.010) 16753184

[28] Fornari S, Schafer A, Kuhl E, Goriely A 2019 Spatially-extended nucleation-aggregation-fragmentation models for the dynamics of prion-like neurodegenerative protein-spreading in the brain and its connectome. bioRxiv. (10.1101/692038) 31809717

[29] Kundel F, Hong L, Falcon B, McEwan WA, Michaels TCT, Meisl G, Estras N, Abramov AY, Knowles TJP, Goedert M 2018 Measurement of tau filament fragmentation provides insight into prion-like spreading. ACS Chem. Neurosci. 9, 1276–1282. (10.1021/acschemneuro.8b00094) 29590529 PMC6014609

[30] Henderson MX, Cornblath E, Darwich A, Zhang B, Brown H, Gathagan RJ, Sandler RM, Bassett DS, Trojanowski JQ, Lee VMY 2019 Quantitative *α*-synuclein pathology mapping and network analysis provide a framework for understanding pathological protein spread. Nat. Neurosci. 22, 1248–1257. (10.1038/s41593-019-0457-5) 31346295 PMC6662627

[31] Betzel RF, Bassett DS 2017 Generative models for network neuroscience: prospects and promise. J. R. Soc. Interface 14, 20170623 (10.1098/rsif.2017.0623) 29187640 PMC5721166

[32] McNab JA *et al.* 2013 The human connectome project and beyond: initial applications of 300 mt/m gradients. Neuroimage 80, 234–245. (10.1016/j.neuroimage.2013.05.074) 23711537 PMC3812060

[33] Szalkai B, Kerepesi C, Varga B, Grolmusz V 2017 Parameterizable consensus connectomes from the Human Connectome Project: the Budapest Reference Connectome Server v3.0. Cogn. Neurodyn. 11, 113–116. (10.1007/s11571-016-9407-z) 28174617 PMC5264751

[34] Dale AM, Fischl B, Sereno MI 1999 Cortical surface-based analysis. I. Segmentation and surface reconstruction. Neuroimage 9, 179–194. (10.1006/nimg.1998.0395) 9931268

[35] Weickenmeier J, Butler C, Young PG, Goriely A, Kuhl E 2017 The mechanics of decompressive craniectomy: personalized simulations. Comput. Methods Appl. Mech. Eng. 314, 180–195. (10.1016/j.cma.2016.08.011)

[36] Jack CR, Holtzman DM 2013 Biomarker modeling of Alzheimer’s disease. Neuron 80, 1347–1358. (10.1016/j.neuron.2013.12.003) 24360540 PMC3928967

[37] O’Dea R, Cofts JJ, Kaiser M 2013 Spreading dynamics on spatially constrained complex brain networks. J. R. Soc. Interface 10, 20130016 (10.1098/rsif.2013.0016) 23407574 PMC3627122

[38] Schafer A, Weickenmeier J, Kuhl E 2019 The interplay of biochemical and biomechanical degeneration in Alzheimer’s disease. Comput. Methods Appl. Mech. Eng. 352, 369–388. (10.1016/j.cma.2019.04.028)

[39] Barthelemy M, Barrat A, Pastor-Satorras R, Vespignani A 2004 Velocity and hierarchical spread of epidemic outbreaks in scale-free networks. Phys. Rev. Lett. 92, 178701 (10.1103/PhysRevLett.92.178701) 15169200

[40] Polanco JC, Bodea LG, Martinez-Marmol R, Meunier FA, Götz J 2018 Amyloid-*β* and tau complexity—towards improved biomarkers and targeted therapies. Nat. Rev. Neurol. 14, 22–39. (10.1038/nrneurol.2017.162) 29242522

[41] Congdon EE, Sigurdsson EM 2018 Tau-targeting therapies for Alzheimer disease. Nat. Rev. Neurol. 14, 399–415. (10.1038/s41582-018-0013-z) 29895964 PMC6463489

[42] Xin S-H, Tan L, Cao X, Yu J-T, Tan L 2018 Clearance of amyloid beta and tauin Alzheimer’s disease: from mechanisms to therapy. Cogn. Neurodyn. 34, 733–748. (10.1007/s12640-018-9895-1) 29626319

[43] Pöschel T, Brilliantov NV, Frömmel C 2003 Kinetics of prion growth. Biophys. J. 85, 3460–3474. (10.1016/S0006-3495(03)74767-5) 14645042 PMC1303654

[44] Chia S, Habchi J, Michaels TCT, Cohen SIA, Linse S, Dobson CM, Knowles TPJ, Vendruscolo M 2018 SAR by kinetics for drug discovery in protein misfolding diseases. Proc. Natl Acad. Sci. USA 115, 10 245–10 250. (10.1073/pnas.1807884115) PMC6187117 30257937

